# Three-Dimensional Stem Cell Bioprinting

**DOI:** 10.16966/2472-6990.110

**Published:** 2016-05-12

**Authors:** Joshuah Gagan, Carolyn Fraze, David A. Stout

**Affiliations:** 1 Department of Electrical Engineering, California State University, Long Beach, Long Beach, CA, USA; 2 Brigham Young University, Idaho, Rexburg, ID, USA; 3 Department of Mechanical and Aerospace Engineering, California State University, Long Beach, Long Beach, CA, USA; 4 International Research Center for Translational Orthopaedics (IRCTO) Soochow University, Suzhou, PR China

**Keywords:** Stem Cell Bioprinting, Embryotic stem cells, Adult stem cells, Neonatal stem cells

## Abstract

Stem cells have become a revived biotechnology that is beginning to expand the field of regenerative medicine. Although stem cells are capable of regenerating tissues, current research trends tend to side on developing fully functional organs and other clinical uses including *in situ* stem cell repair through three-dimensional printing methods. Through several tests and techniques, it can be shown that most stem cell printing methods are possible and that most tests come out with high cell viability. Furthermore, the importance of bioprinting is to benefit the field of regenerative medicine, which looks into artificial organ transplants for the thousands of patients without donors. Although the field is not brand new, understanding the integration and use of additive manufacturing with biomaterials is essential in developing fully functional organs. There is a heavy emphasis on the biomaterials themselves since they have a crucial role in creating an organ that is mechanically robust and adaptable *in vivo*. Covered in this review article are many featured tests, which also touch on the importance of including a biomaterial that is capable of maintaining a viable microenvironment. These include biomaterials such as hydrogels, biopolymers, and synthetic extra cellular matrices (ECM) built for stem cells to proliferate, differentiate, and give freedom to cell communication after printing.

## Introduction

From the late 1990s, Stem cells have been in speculation for their potential to differentiate into multiple types of cells and their self-renewal [1]. However, at the time they were discovered, the only type of stem cell available was Embryonic Stem Cells (ESCs). To obtain viable ESCs, researchers had to complete many daunting tasks and mix political perceptions with research, which made conducting research very challenging [2]. Due to this, stem cell research was delayed and conflict of interest rose. In later years, regenerative medicine came on to the scene. This revived interest in stem cell research and knowledge of stem cells grew rapidly [3,4].

Around the same time, additive manufacturing was also in demand, with speculation on the ability to create industrial products out hardened Polyactic Acid (PLA) [5]. What made it so appealing to clinical researchers was its precision, which would open opportunities to reduce error in creating three-dimensional scaffolds by hand. Researchers from across the globe began testing stem cells and biomaterials together in an attempt to develop one of the first artificial organs [6].

This effort was achieved in 2009 and soon after bioprinting was made possible [7]. In short, the technique is simple: biomaterials are allotted into a tubule, which is then pressurized and pushed out to form a tiny droplet. All of these droplets are formed instantaneously upon contact with the printer bed.

The mechanics of additive manufacturing work well, but there is one problem: cells are living organisms. They are not stationary and have a tendency to migrate naturally. They may also die if the printing method destroys their microenvironment or if their microenvironment is not stable. Additionally, if stem cells are not as responsive to work with the biomaterial it is printed with, then the stem cells may migrate to certain areas of the print and create abnormalities in larger scale tissues [8]. In order to master bioprinting, understanding the properties and behaviors of stem cells with biomaterials are necessary.

### Stem Cell Types

There are now more than a couple different types of stem cells and featured here is their ideal properties to be put to use in three-dimensional printing methods to create living tissue and organs. Moreover, the most essential part of printing cells is how they will react to it.

**Figure 1:**
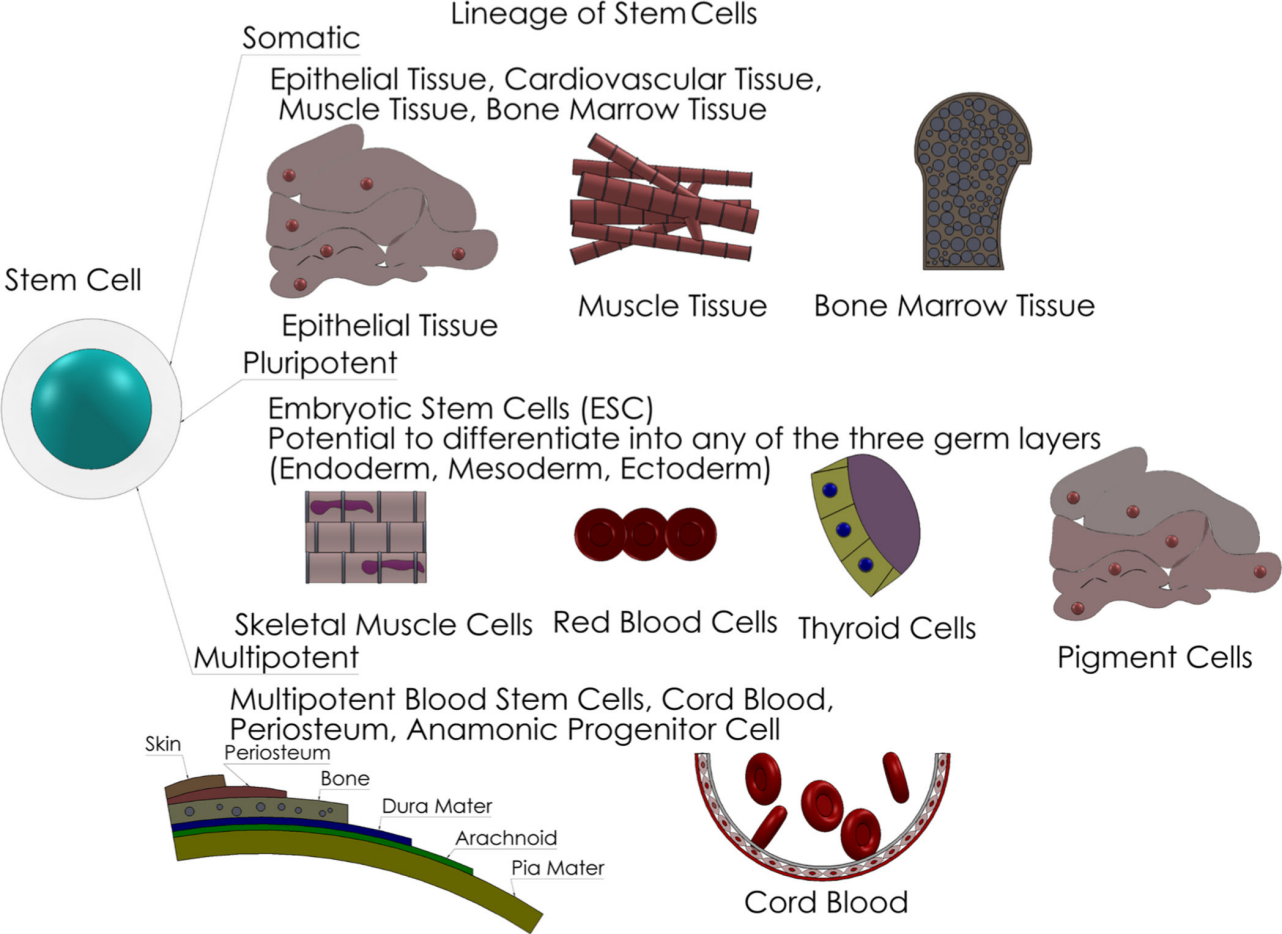
The most common stem cell types. Each one has their own unique pathway and includes several possible descriptions. A) Somatic Cells, also known as adult stem cells, do not differentiate into a different type of cell. Rather, they stay among the cell groups inside of the niche it is held in. It is capable of regenerating and reviving specific tissues it is grouped in. Somatic stem cells can be found in many places in the body, depending on the niche location of each tissue. B) Pluripotent Stem Cells are stem cells that can differentiate into any type of cell and will thrive in any type of tissue. Embryonic Stem Cells are the most common in this category. These cells have certain properties that can generate the three germ layers of the cell. Most of these are found in neonatal material or embryos C) Multipotent Stem Cells are similar to pluripotent stem cells, but to renew itself it must differentiate into a different type of cell. This specializes them and their self-renewing property is makes them capable of producing a few more cycles. Multipotent stem cells are more commonly found in bone and cord blood.

To start, it is necessary to look at what types of stem cells are capable of undergoing the additive manufacturing process. Most stem cells are capable of being extruded. The following stem cell types have been studied in numerous labs and are displayed here figure 1 to show the variety and difference of each one.

### Embryotic stem cells

When scientists first discovered ESCs, they were astonished to find they had the potential to solve certain ailments and diseases. In contrast, ESCs would be difficult to harvest in mass quantities. These cells are only available during embryonic fertilization where they differentiate into the design of the human anatomy [9]. What makes these special, in particular, is their ability to differentiate into almost every cell type [10]. Embryonic stem cells need no former parent lineage to match the desired cell, reducing the need for proteins or agents to morph the cell.

What makes ESCs unique and different from most other stem cells is the ability to create three different germ layers: endoderm, mesoderm, and ectoderm. Each one of these layers accompanies the stem cell and gives the multipotency factor that makes the cell universal. Per layer, the germ layers act as influencers to differentiate the cell into a separate lineage [11,12].

### Adult stem cells

Derived from adult tissue, adult stem cells (ASCs) come from certain areas of tissue that serve as a niche and are released during an injury to rebuild the tissue necessary to re-growth [13,14]. Also known as somatic stem cells, each ASC can only differentiate into their parent lineage and their respective cells. That does not put a limit on stem cells but it does affect their ability to regenerate into their former cell type. Some of the most common areas of ASCs are in epithelial tissue, cardiovascular tissue, muscle tissue, and one of the most popular is bone marrow tissue [15]. For bone marrow stem cells (BMSCs), they are widely regarded as one of the easiest to use [16]. Many research groups have taken advantage of using them to develop three-dimensional printers as bone is one of the simplest tissue types in the body made of calcium, fat, and blood/plasma [17].

### Neonatal stem cells

After childbirth, the remainder of the uterus contains the rest of the umbilical cord and amniotic fluid. These are not hazardous wastes, as most of the material is neonatal. A large portion of this waste contains stem cells that are alive and culturing, in part of creating the child inside the womb [12]. In fact, neonatal waste is donated to a public/private bank for cryopreservation and examination to harvest the stem cells that have been derived from the womb. The stem cells derived from it are almost a sister to ESCs [12]. Neonatal stem cells (NSCs) have abilities similar to ESCs, including regeneration and pluripotency. Yet, what limits their use in the field of regenerative medicine is that they are autologous, meaning they can only be used on the individual they came from [18].

### Human-induced pluripotent stem cells

Another relative to ESCs, human-induced pluripotent stem cells (hPSCs) which are derived from somatic, terminally ill cells that have been reprogrammed to function as ESCs [19]. The current methods of reprogramming are almost new, but the cells act as they are told to be.

The limitations on hPSCs are the limit in gene expression [2]. Research has yet to detest the effects of reprogramming and how they act between natural cells instead of reprogrammed cells [20].

There is one factor that matters the most and that is how to cultivate stem cells into abundant quantities. As of yet, no efficient way has come to light, since stem cells are capable of differentiating into an undesired cell depending on the conditions they were raised in.

The same could be said about similar cell types as well, depending on the multipotency of the cell itself. Some stem cells, including ASCs are only capable of differentiating into their parent tissue [10]. If epithelial adult stem cells were taken and cultured, they would regenerate only into epithelial cells. In some cases, morphogenic proteins have been studied with stem cells to influence them to grow into other cells unlike their parent source [21].

Influencing a new protein into the cell culture has numerous effects. One study on multipotent adult bone marrow-derived mesenchymal stem cells (MSCs) is experimenting for use in develops different tissue types using Bone Morphogenic Protein-2 (BMP2) [22]. In this case, BMP2 classified the cells into three different lineages: cartilage, renal, and epithelial. All three were situated in a certain environment that had materials in the surrounding matrix with each respectively influencing the cells to the appropriate lineage. The cells are able to know thanks to BMP2, as it serves as a communicator for cell signaling and cell environments. This protein gives the guidelines and knowledge for stem cells to differentiate into the desired cell and develop tissue based on the experiment conducted.

It would be ideal to control cell signaling between stem cells, primarily to grow in abundance for the use of additive manufacturing.

### Stem Cell Microenvironments

Research has shown that four factors need to be addressed when developing a stem cell microenvironment: cell migration and movement, environment remodeling, change in phenotypic expression, and cell viability. Each plays an important role in controlling a stem cell and the reactions that it may have in an engineered microenvironment should not be treated as a material, but a living organism [23,24].

Despite success with BMP2, stem cells cannot rely on cell signaling alone to maintain homeostasis. In some cases, they may not find the environment they are placed in suitable which could lead to cell differentiation or cell destruction [22,25,26]. One way to reduce cell destruction is in microencapsulation, which surrounds the cell in an extra-cellular matrix (ECM) environment to provide proper nutrition, hydration, and accessibility to communicate with other cells.

The most popular method is by encapsulating stem cells in hydrogels, which contain all of the resources necessary to keep stem cells intact and undifferentiated. Hydrogels themselves provide an atmosphere suitable and ideal because they are porous, made of water, and biodegradable [24]. Most hydrogels are made of organic material, some of which are polysaccharides like alginate or proteins such as collagen and fibrin [27]. All of these become hydrogels by creating a rigid shape for the addition of water molecules or a type of liquid to enter. Cells are then encapsulated into the material and begin to retain homeostasis by adjusting to the new environment [23,26].

However, if the stem cells cannot adjust to their new environment, they will modify the environment. It comes down to what is needed inside of the ECM they are introduced to and how it effects them. Hydrogels may seem ideal, but it may not hold a rigid, mechanically robust structure. This technique works well with for single stem cell printings on cells and tissue; full-scale organs may not find them beneficial [25]. The intricacies of organs themselves may make it complicated for hydrogels alone to create a structure so precise. If all else fails, they will release particles into the matrix to develop their own habitat, some of which are basic proteins for cell survival. This is all for the stem cell to adapt to its new environment and ensure its self-assurance for survival.

### Biomaterials

Once stem cells are homeostatic in the environment they are placed in, they have the potential to be used to develop tissues and even organs [28,29]. Although hydrogels with encapsulated stem cells cannot create tissue, there are biopolymers or biomaterials that serve as a compliment to create a rigid, self-standing object. Biopolymers range from a wide variety of materials [27].

For this to occur, specific qualifications for creating a supplemental biomaterial need to be addressed; cell adherence, low toxicity, biodegradable, and permeable. For example, when applying hydro gel micro beads onto the biomaterial, it is good to ensure that the structure itself will not fall apart. Some adhesives on biomaterials will connect to the hydrogels using chemical properties or sometimes through the cells themselves [23].

For cell scaffolding, this is an easy process. The cells attach themselves to the biomaterial and then when implanted *in vivo* slowly take over and expand across the material it is scaffold with. The process works for bone and hard cartilage tissue, but as for fully-functional organs, there should not be any scaffolding.

Currently, researchers are looking forward to creating scaffold-free organs which would involve making the stem cells dominant and possibly take over the entire structure to degrade it down [10]. That way, the artificial organ would have only tissue around it and not biopolymers.

There are not too many drawbacks to taking this method. For one thing, scaffold-free organs are similar to methods in tissue engineering [30]. In tissue engineering, stem cells are cultivated and seeded onto a 3D scaffold made by the researchers themselves although now it could be printed using additive manufacturing. Once cell cultivation grows a sufficient amount, they are seeded onto the scaffold and the cells attach themselves with one another to engulf the scaffold. The cells adjust, decompose the biomaterial and begin to form the desired shape of the organ and most likely begin filling in the functions of the organ itself [31].

### Polyactic acid

Polyactic acid (PLA) has been shown to assist industrial use than for clinical. Yet there is a need to take notice that PLA is not a synthetically manufactured, but rather naturally grown [5]. PLA is derived from cornstarch and other such plants, purified and composited into a filament for traditional extrusion [32].

Even though PLA is not generally used for biomaterials, it is for one thing biomimetic. Its material is mechanically robust and is made of natural resources which could hold hydrogels or similar microencapsulating gels. The toxicity level on PLA is low as well [5]; bearing in mind that cornstarch is not one to have too many toxins in it [2].

As far as permeability is concerned, it has recently come to limelight [23]. As a cell, it must be able to transfer nutrients and protein synthesis as well as waste materials across its semi permeable membrane [23]. Unfortunately, researchers cannot use PLA due to its porosity as the membrane would inhibit the waste inside of the material and could transfer over to the body.

### Alginate

Formed from red algae, alginate is almost a gel but its sol-gel mechanism creates it to be a mechanically robust structure. It also has a wide pore distribution, allowing materials to go through a concentration gradient at the stem cell’s discretion. Unfortunately, alginate is not biodegradable or adhesive. The material itself does not have the chemical composition to hold onto other materials or the ability to allow biodegradation.

Fortunately, there is a loophole around the methods of using alginate with stem cells, which can explain why it has been used clinically for so long [23,31]. Alginate is a rare biomaterial and to keep its properties without compromise, agents can be used to add additional features. For cell adhesion, collagen can be mixed with alginate. Collagen is another natural biomaterial that is derived from ligaments and skin. Thanks to its elasticity and triple helix structure, it connects with other soluble materials to itself [23]. This links with the alginate and the hydrogel that is applied onto it for stability.

As far as biodegradability, *in vivo* still remains as a problem. That is not to say biodegradability *in vitro* will not be. An agent called Ethylenediaminetetraacetic acid (EDTA) is applied onto the alginate substrate. Over time, it begins to breakdown the internal structure that formed the shape of the scaffold to allow stem cells to overwhelm and take its shape [27]. By being able to find a biomaterial that supplements the structure of the desired tissue, biomaterials have the ability to design fully functional tissues and even organs.

### Hyaluronic acid

Probably the most ideal, hyaluronic acid (HA) has what almost any biomaterial would need. The polysaccharide is natural, stemming from a variety of different organisms [23]. It is biodegradable and allows other organisms to attach to it thanks to its RGD adhesive [23]. HA is a compatible biomaterial and could be used over alginate as it serves to be the opposite of what alginate does not have.

Unfortunately, there are a few things that make the material different. The effects of HA *in vivo* and *in vitro* are a bit different [24]. The water content in HA is higher than most biomaterials and could lyse some of the cells upon attachment or encapsulation [23]. However, the degradation rate is lower than most biomaterials, making it less susceptible of giving dominance to stem cell cultures that would take over the biomaterial post-printing. The effects of said properties would not be so drastic *in vitro*, but may have long-term effects to the metabolic processes in the body.

### Reactions of Stem Cells and Biomaterials

Knowing that biomaterials have the potential to create a temporary scaffold and proper microenvironment, what matters the most is how combining both a hydrogel and a biomaterial together would give the cells freedom to develop tissue. Unfortunately, it all depends on the situation alone. Stem cells can grow their own viable scaffold on their own if the tissue is epithelial and injected *in situ* [15]. In demonstration, this almost replicates the methods tissue engineers use to recreate epithelial tissue by simple micropipetting and drop-on-demand [33,34].

Once the cells are placed onto the area, they begin to recognize the environment they are placed on and begin differentiating into the appropriate tissue. For example, the cells are simply injected, leaving their new environment to do the rest. On the other hand, stem cells may be combined with a heterogeneous mixture of biomaterials reminiscent to a gel [27]. This provides an ideal ECM and encourages cellular growth.

When using this method, there was a significant advantage as opposed to using stem cells alone [23,33]. In the same lab where stem cells were injected *in situ* onto open wounds, stem cells and a couple gels (one of them being alginate) had an increase in cell migration [15].

Although this was not expected as the purpose was to demonstrated the gel’s mechanical robustness, it highlighted that cells injected in another biomaterial increase cell migration, as the tissue treated went from 42% damage to 3% damage within two weeks [15]. The cells formed a tight bond with one another and then formed naturally into the epithelial layers.

Besides tissues, there is a need to develop three-dimensional and fully functional organs. The method here proves that biomaterials work well with stem cells and promote cell growth, but does not prove how structures remained intact [35]. Efficient structure and rigidity are major functions in developing artificial organs.

Some methods rely on structures embedded into the biomaterials themselves for the cells to grow around18. That is a possibility and may work for creating the intricacies of most organs like kidneys [14,36]. It is also important to note cell positioning. There is not much consideration where a cell is encapsulated into or where it is placed. Research has noted that there are variations on where a cell might have the best or worst results based on their location [21,37,38].

Primarily, the focus is on cell differentiation and spatial distribution to get stem cells to their fullest potential. This mainly has to involve with the cell culture that takes over the biomaterial-scaffold, which will have a later effect on how the cells interact with each other. For example, if an organ was to be bioprinted, there are different tissue types that will be used. Each stem cell should not become the same cell type but rather a variety of cell types.

In addition, the spatial distribution is dependent on the exact area on where each tissue resides of that respective organ. Within the printing process, the biomaterials may also contain agents or protein that would influence particular differentiation. All of these factors are crucial to making all of the working systems of the tissue synchronize together so that the tissue itself performs seamlessly.

### Bioprinting Methods

**Figure 2:**
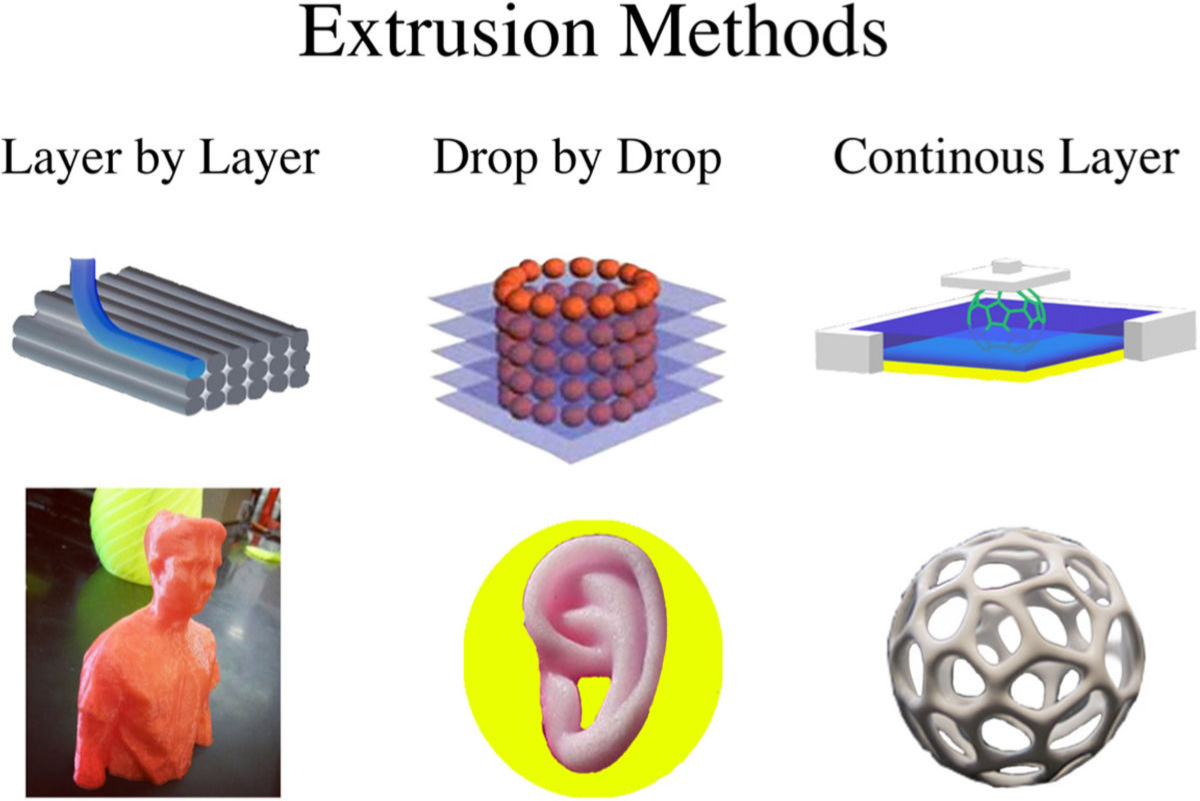
Extrusion three-dimensional printing methods and the prints that they have produced. Layer by Layer extrusion is a traditional approach to creating a three-dimensional object. It divides the object into several cross sections and adds a layer into the appropriate cross-sectional area. Inkjet printing and extrusion-based printing utilize this method and have the most precision. Drop by Drop extrusion is almost comparable to scaffolding with cells. During the printing process, a scaffold is generated through a flexible biomaterial onto the printing surface. As it is printing, an additional nozzle extrudes nanoparticles that contain cells. Once the biomaterial is placed onto the printer bed, the droplet nozzle carefully places cells onto the object to create a cell culture. Continuous Layer Extrusion is a new method that has not been fully studied yet to its potential. Essentially, a plate slowly rises above a viscous liquid. As it escalates, the material is photopolymerized by UVA light from beneath the plate. As photopolymerization occurs, cross-sectional layers are made and keep their rigidity, which keeps it stuck to the plate until the object is fully extruded.

When investigating bioprinting, three factors that need to be addressed when stem cells and biomaterials dispensed from three-dimensional printers are: (1) forces acting on the materials and dispenser, (2) timing of the dispenser, and (3) cell deposition. All of these are critical to the success of building organic tissue or artificial organs. Yet, there are many ways that have been tested to create tissue using modified printers, extruders, or even lasers [28,29,35]. The following discusses more in-depth on the advantages and disadvantages of printer methods that have been used for experimentation (Figure 2).

### Extrusion

One of the most common and made for industrial purposes, and most preferred is three-dimensional extrusion [7,32,39]. By using a piezoelectric pressure valve and a xyz-axis, extrusion accepts most biomaterials and stem cells to create a layer-by-layer deposition. As the object is created, the user has the ability to control the extrusion speed, thermodynamic properties, and cell placement.

Creating the tissue itself is also efficient, too. Thanks to CAD/CAM software [40], a tissue or organ can be scanned with computerized tomography (CT) to generate the blueprints and target points to where the cells will be positioned [32]. Timing is also appropriate, as the user is able to control when the cells will be placed. This gives an advantage to sol-gelation, if hydrogels are to be extruded through the material so that they are not dehydrated when placed atop of the printer plate.

There is some debate in the extrusion process, as most extruders are paired with a hot-end and plate is essentially a heat-bed [32]. With client software, users are able to control if thermodynamics need to be used or not. Should the materials be cryogenically frozen depends on the experiment and the effects of cryo-freezing on stem cells. There is an option to disable the thermodynamics to store cells and use them at a later time (biomaterials won’t need to do so) [41].

In addition, there is also a concern on the shear forces of the piezoelectric extruder. As a material is being dispositioned, it goes through the extruder to form a viscous droplet. In doing so, there are forces that enact onto it to make the materials rigid and precise so that it does not sputter onto the plate [34,42]. However, this can be a risk as some cells are recorded to decrease in viability after extrusion. Additionally, there should be enough cells to engulf the structure the biomaterials formed, but that has yet to be proven with more statistical data from other research groups.

In the end, there are modifications that can be done to the extruder, some of which can dispense high viscosity materials. The newfound micro extruder, which mimics micropipetting is able to generate more precise prints and gives the advantage of recreating the intricacies of organ-specific tissue functions [8,27] Scientists have also developed a piezoelectric microfluidic chip [34], which borrows the idea of micropipetting and reduces contact forces between the plate and extruder.

### Inkjet printing

Similar to extrusion is the traditional inkjet printing. The mechanics of a paper printer are the same, but instead of using ink and toner, bioinks are in place of the cartridges [43].

Instead of using a plate, a solvent is sprayed onto the base where the prints are to be dispensed on [41]. Inkjet printing has a small difference from extrusion. Instead of releasing materials one at a time, the bioink is sprayed over to a specific shape and pressed to form the geometric shape designed. The solvent crosslink’s the bioink to the structure to form the designed shape. Tissues and cells have been successfully printed from this process.

Probably the most significant piece of inkjet technology is the bioink, a heterogeneous fluid made of cells, proteins, and fluids to hydrate the microenvironment. Inkjet technology was widely used in the mid-2000s, before the age of three-dimensional printing extrusion. Before then, Dr. Atala [44] developed a way to create heart valves using inkjet printing technology, which inspired several scientists to try the same method.

The advantage of inkjet printing for biomaterials are similar to that of extrusion, where prints can be controlled by will of the user and guided by office software. Unlike extrusion, the prints avoid direct contact on the plate, rather a solvent to guide the cells to migrate. The probable disadvantage is cell lysing [35,41]. It does not have a dramatic effect on the cells while being printed, but the concentration of fluid acting onto the cell membrane can cause the cell to burst. Nevertheless, in a recent study, approximately 10% of cells lysed [41]. The cause could be directly related to the number of hydrogels that were in the bioink.

### Laser-assisted printing

The most complex of all printing methods, laser-assisted printing is a different realm of printing. The method start with are two glass slides, one being the donor slide that has the encapsulated cells in hydrogels and the collector slide which contains an additional hydrogel layer to reduce the impact of the laser energy transfer [35,45]. When the laser is activated, it shoots into a gold-film that covers the cells and prevents them from being destroyed. This energy starts to absorb the hydrogels on the donor slide and transfers them over to the collector slide. The cells are transferred to their specific place based on the user’s CAD/CAM design and deposited.

Ironically, lasers may have no effect on the cell viability [11,45]. As the cells are absorbed the gold plating acts as a protective shield for energy absorbance and placed as designated [11]. It does introduce a new technology and focuses more on cell absorbance through laser energy.

### Photopolymerization

Similar to laser-assisted printing, photopolymerization utilizes light energy to create objects [46]. The materials are placed into a resin bath and covering it is the plate. The object is printed using UVA light that photopolymerizes the materials depending on their cross-sections. Wherever the UVA hits the material hardens and while UVA is flashed the base plate lifts itself up to display the object [5].

The biggest advantage is the printer’s ability to absorb light energy and harden cells fast and the process is quicker than most conventional three-dimensional printers [5,47]. The drawback to the printer’s method is the UVA radiation. If the radiation is strong enough, stem cells inside may differentiate and become carcinogenic. In one test, researchers measured the affects of UVA radiation on cells using a cytotoxicity test in the course of three days [46]. Over time, researchers found that most cells did die over the process only to self-renew after the cells’ “lag phase.” [46]. Eventually stem cells started to culture and overpopulate the biomaterial for degradation.

**Figure 3:**
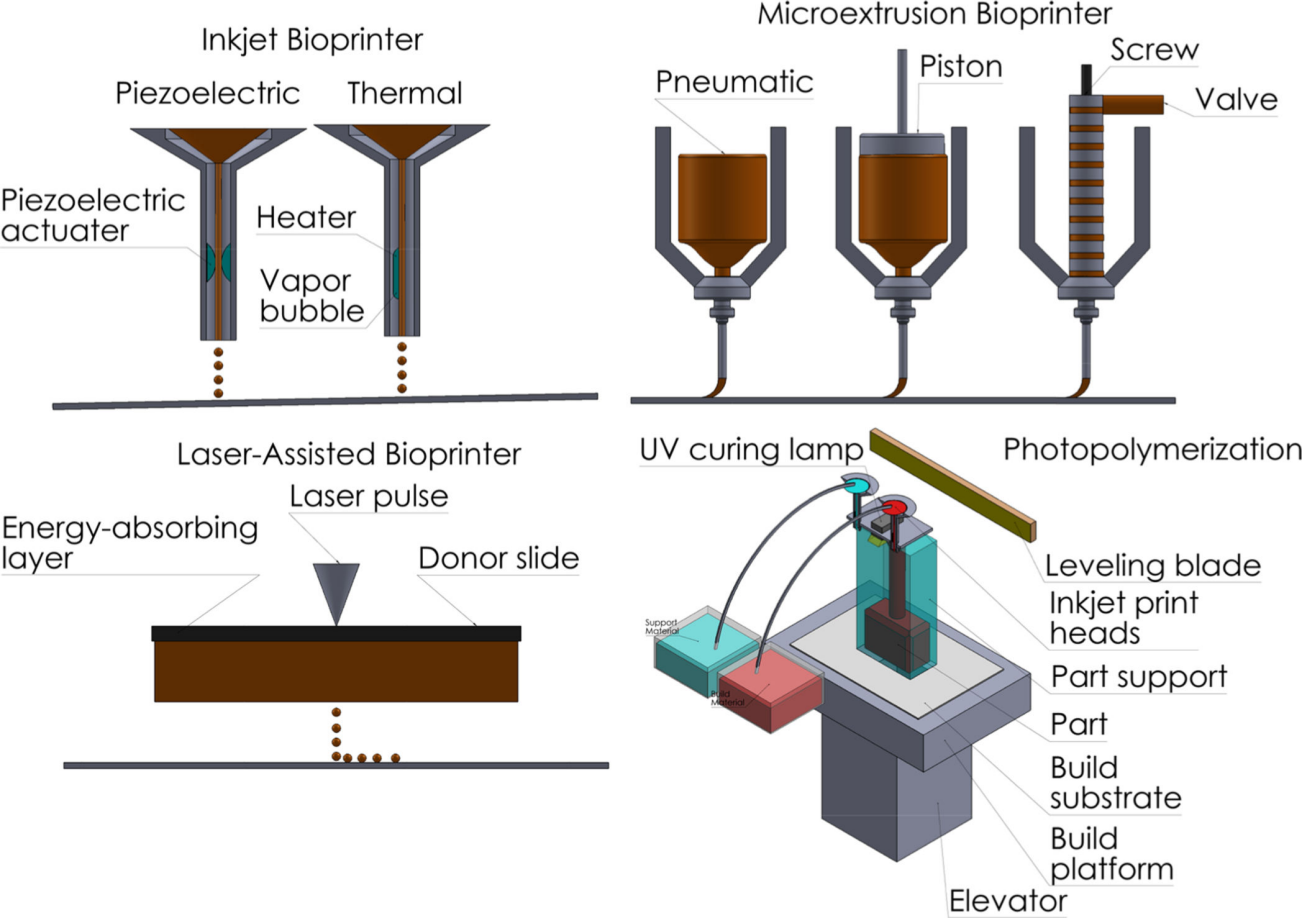
For each extrusion method, there are different forces and techniques applied per extruder. No extruder is deemed the same way, since each one is specific to the biomaterial printed. Inkjet Bioprinters can use a piezoactuator to apply forces for fast extrusion or thermal heat to melt the material as it drips down to the surface in a concise manner. Microextrusion is a more specific extruder than inkjet bioprinters. Since the materials are much smaller, different forces can be used to dispense the material. These range from pneumatic, screw, and piston. Laser-Assisted Printing is unique to those of the previous to extrusion methods. By using a laser, particles are guided from an energy absorbing layer to the donor slide. During this transition, particles accumulate onto the slide and are not damaged by the laser’s

The biggest drawback was that this was designed only for a two-dimensional microenvironment. Cells are required to thrive in a three-dimensional microenvironment that would model *in vivo* conditions. As it was not the case, this method would not be relevant towards the use of creating tissues and organs (Figures 2 and 3).

### Considerations

Regardless of which method is chosen, it is safe to conduct a three-dimensional stem cell tissue or organ. Each method does have certain advantages and drawbacks, but will give the best-produced result from their system (Table 1). However, there are a couple points to consider as to how these models are designed and created. When a stem cell overpopulates the biomaterial, it degrades to give rise to the new tissues naturally.

However, after the microenvironments the cells were once present in are destroyed, how will the new tissues work as a system together? This is what researchers call vascularization, the process in which vascular systems are introduced into the system. This includes veins, capillaries, and other blood pathways that filter and transfer materials into tissues. It is not certain that a stem cell environment is capable of recreating a vascular system, as the cells are designed to differentiate into the tissue cell type.

So far, only a few methods have been published on the vascularization of artificial tissues [31,47] with one of them includes artificially creating the vascular system by hand. As the materials are deposited onto the printer base, vascular grafts and capillaries are placed below the extruder to be enveloped by the new material it will grow around. Once the biomaterials degrade, the idea is to get the stem cells to differentiate not only into the tissue cell type but into the vascular type as well. This may involve the need to create a separate biomaterial that influences differentiation into vascular grafts. Much has yet to be said about the subject.

Another consideration is the difference between the effects of *in vitro* and *in vivo* environments for stem cells. On one hand the stem cells may appear to differentiate as planned post-printing and may seem acceptable for transplantation.

Yet, there are scarcely any findings on the effects of transplantation *in vivo*. Dr. Atala [44] at Wake Forest University demonstrated it in 2011 on a student named Luke, who received an artificial liver transplant created *in vitro* by an inkjet printer. Luke is currently alive and well today, and has not experience any terminal problems so far. Dr. Atala’s method is briefly published, so it is not certain as to what materials or cells he did use [6,7,10].

Modeling an environment that mimics the homeostatic functions of the body is the best way to determine the functionality of artificial tissue. It would dramatically reduce the risk of mammalian testing and risk of implantation. A model has yet to be found, one that would stimulate vascularization, metabolic processes, and immune reactions if the body undergoes an infection. All things considered, these are just a few of what would simulate the environment.

Lastly, there is still a need to consider the effects of stem cells and their possibility to differentiate into unwanted cells. The printing methods aforementioned can destroy cells, but in some cases stem cells can become carcinogenic. For example, UVA radiation in photopolymerization printing can create a tumorigenic cell type and stem cells could overpopulate into a similar lineage, thus destroying the tissue and ruining the model. The same could be said for extrusion, depending on the user’s choice for thermodynamics. The hot end of the extruder can reach temperatures of up to 200C which could cause a negative reaction in the stem cells during extrusion. In the end, stem cell printing has a few things to improve on. The methods used are sufficient, but some intricacies need to be improved on.

Regardless of which method is chosen the most critical thing to understand is the use of stem cells in a microenvironment and the biomaterials that compliment them.

## Figures and Tables

**Table 1:** Analysis of Bioprinting Methods with Biomaterials and Stem Cells

Stem Cells	Bioprinting Techniques	Outcome	Purpose	Reference
Human Adipose Derived Stem Cells	Laser Assisted Bioprinting	No significant difference in Proliferation. Adipoblasts were made into Lipid Molecules. All cells were viable	To create artificial skin grafts from adipogenic cells.	[45]
Human Pluripotent Stem Cells	Modified Piezoelectric Extruder	Pluripotency Property remained unchanged postprinting. Hepatocyte markers indicated possible differentiation. Cell Viability was between 72%−96%	Demonstrate Valve length and the creation of mini-livers utilizing pluripotency	[19]
Multipotent Stromal Cells	Photopolymerization using UVA Light	Based on the proteins linked, several cell types were derived. Cell viability was at most 90%	To develop a variety of osteogenic cell lineages and to demonstrate the use of UVA light to print out materials	[46]
Human Adipose Derived Stem Cells	Inkjet Bioprinting	Polymers with encapsulated cells remained intact. No info on cell viability besides success in cell cultures	Show the mechanical robustness and rigidity of the polymers with the cell cultures that follow	[37]
